# COVID-19: cross-border contact tracing in Germany, February to April 2020

**DOI:** 10.2807/1560-7917.ES.2021.26.10.2001236

**Published:** 2021-03-11

**Authors:** Inessa Markus, Gyde Steffen, Raskit Lachmann, Adine Marquis, Timm Schneider, Sara Tomczyk, Uwe Koppe, Anna M Rohde, Susanne Barbara Schink, Janna Seifried, Teresa Domaszewska, Ute Rexroth, Maria an der Heiden

**Affiliations:** 1 Robert Koch Institute, Unit for Surveillance, Department of Infectious Disease Epidemiology, Berlin, Germany; 2 Robert Koch Institute, Postgraduate Training for Applied Epidemiology (PAE), Berlin, Germany; 3 European Programme for Intervention Epidemiology Training (EPIET), European Centre for Disease Prevention and Control, Solna, Sweden; 4 These authors contributed equally to this work; 5 Robert Koch Institute, Unit for HIV/AIDS, STI and Blood-borne Infections, Department of Infectious Disease Epidemiology, Berlin, Germany; 6 Robert Koch Institute, Unit for Gastrointestinal Infections, Zoonoses and Tropical Infections, Department of Infectious Disease Epidemiology, Berlin, Germany; 7 Robert Koch Institute, Unit for Immunization, Department of Infectious Disease Epidemiology, Berlin, Germany; 8 Robert Koch Institute, Unit for Healthcare-associated Infections, Surveillance of Antibiotic Resistance and Consumption, Department of Infectious Disease Epidemiology, Berlin, Germany; 9 Robert Koch Institute, Department of Infectious Disease Epidemiology, Berlin, Germany; 10 Robert Koch Institute, Unit for Respiratory Infections, Department of Infectious Disease Epidemiology, Berlin, Germany

**Keywords:** pandemic preparedness, contact tracing, cross-broader, International Health Regulations, Early Warning and Response System (EWRS) COVID-19, SARS-CoV-2, Germany

## Abstract

**Introduction:**

The Robert Koch Institute (RKI) managed the exchange of cross-border contact tracing data between public health authorities (PHA) in Germany and abroad during the early COVID-19 pandemic.

**Aim:**

We describe the extent of cross-border contact tracing and its challenges.

**Methods:**

We analysed cross-border COVID-19 contact tracing events from 3 February to 5 April 2020 using information exchanged through the European Early Warning Response System and communication with International Health Regulation national focal points. We described events by PHA, number of contacts and exposure context.

**Results:**

The RKI processed 467 events, initiating contact to PHA 1,099 times (median = 1; interquartile range (IQR): 1–2) and sharing data on 5,099 contact persons. Of 327 (70%) events with known exposure context, the most commonly reported exposures were aircraft (n = 64; 20%), cruise ships (n = 24; 7%) and non-transport contexts (n = 210; 64%). Cruise ship and aircraft exposures generated more contacts with authorities (median = 10; IQR: 2–16, median = 4; IQR: 2–11) and more contact persons (median = 60; IQR: 9–269, median = 2; IQR: 1–3) than non-transport exposures (median = 1; IQR: 1–6 and median = 1; IQR: 1–2). The median time spent on contact tracing was highest for cruise ships: 5 days (IQR: 3–9).

**Conclusion:**

In the COVID-19 pandemic, cross-border contact tracing is considered a critical component of the outbreak response. While only a minority of international contact tracing activities were related to exposure events in transport, they contributed substantially to the workload. The numerous communications highlight the need for fast and efficient global outbreak communication channels between PHA.

## Introduction

Since January 2020, severe acute respiratory syndrome coronavirus 2 (SARS-CoV-2) has rapidly spread to become a global pandemic [1]. Active case finding, early detection and isolation of cases and their contacts are essential for breaking transmission chains. A modelling study showed that 70% of contacts should be traced in order to control the outbreak, assuming a baseline reproduction rate of 2.5 [2].

Early warning systems for the serious cross-border spread of infectious pathogens include the International Health Regulations (IHR) 2005 and the Early Warning and Response System (EWRS) for the European Union/European Economic Area (EU/EEA) countries [3,4]. Within Germany, communication channels have been established in accordance with the German Infection Protection Act (Infektionsschutzgesetz; IfSG). Cross-border contact tracing at the national level is operated by the Robert Koch Institute (RKI), the federal public health institute in Germany.

The first cases of coronavirus disease 2019 (COVID-19) in Germany occurred in Bavaria at the end of January 2020 [5]. The first SARS-CoV-2 cluster also led to cross-border contacts and exposures on flights since close contacts and suspected cases travelled to Austria and Spain after exposure. This required intensive international communication to identify and share the information on contacts with the responsible health authorities. An international communication and contact tracing team (RKI IC-Team) was rapidly created in the RKI COVID-19 Emergency Operations Centre (EOC) including members of all units of the department for infectious disease epidemiology and other departments at the RKI. The core task of the team was to collect and communicate information on confirmed COVID-19 cases and their contacts to other countries in the event of cross-border relevance. In addition, incoming information on German citizens exposed abroad was communicated through the federal state health authorities to the responsible local health authorities in Germany.

The spread of SARS-CoV-2 in Germany triggered the introduction of various measures: (i) mass gatherings with more than 1,000 participants were banned after calendar week 10, (ii) schools and public places were closed in several federal states, (iii) physical distancing measures of at least 1.5 m to another person were recommended, (iv) it was recommended to cancel non-essential travel and (v) quarantine measures for travellers from high risk areas entering Germany were introduced in calendar week 15. Because of the federal structure in Germany, the measures and their implementation varied between the states.

This work aimed to describe the extent and course of activities resulting from information on COVID-19 exposure events with a cross-border context. Further, we discuss the challenges experienced and possible workflow improvements.

## Methods

### Information flow in the context of international contact tracing

Information on cross-border COVID-19 exposure events and personal data were shared between the RKI and EU/EEA countries via the EWRS communication platform which provides a single-window messaging system (so-called selective exchange) to communicate with EU/EEA countries and transmit personal data securely. The World Health Organization (WHO) member states outside the EU/EEA received information through the IHR National Focal Point (NFP) by email. In Germany, the IHR NFP is located in the Joint Information and Situation Centre of the federal government and the federal states. Within Germany, email or telephone was used to communicate with health authorities. Personal data were transmitted using an encrypted exchange server (Cryptshare).

**Figure 1:**
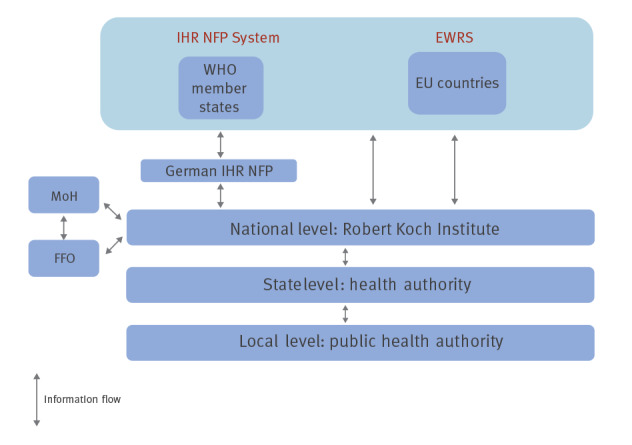
Flow of information in the context of contact tracing in Germany and abroad

EEA: European Economic Area; EWRS: Early Warning and Response System; EU European Union; FFO: German Federal Foreign Office; IHR NFP: International Health Regulations (2005) National Focal Point; MoH: German Federal Ministry of Health; WHO: World Health Organization.

Upon receipt of information, the RKI IC-Team assessed the content and determined the required action. The COVID-19 case and contact person definition employed by the country transmitting the data was used accordingly. The information was forwarded through the federal state health authority to the respective local public health authority (PHA) where the contact was living or currently staying. The local PHA then proceeded with contact tracing activities (i.e. telephone interview, regular monitoring) according to the IfSG. Similarly, information from the local PHA concerning persons staying abroad during the infectious period was forwarded to the responsible foreign health authorities (Figure 1).

In addition to the local German PHA and PHA from foreign countries, the RKI was in contact with the German Federal Foreign Office and its embassies abroad.

### Logistical considerations

The RKI IC-Team worked in two shifts per day. Outside office hours, an on-call epidemiologist was available. Standard operating procedures (SOPs) were developed to enable the continuity of work, and team members were regularly trained and regular opportunity for feedback was provided.

### Data management and protection

Each incoming COVID-19 cross-border exposure event received an internal identifier (ID) to which all information was assigned. The ID was documented in a line list including the date of the receipt of the initial communication, the start of RKI activities, the last possible exposure date of the contacts to a confirmed COVID-19 case, a description of the exposure, the affected countries, the number of contacts and an activity log for all communications. All personally identifiable information on cases and contacts was stored in a separate secure drive in accordance with the General Data Protection Regulation (Directive 95/46/EC) [6]. When the outbreak activities are completed, this information will be permanently deleted.

### Inclusion and exclusion criteria

We reviewed all COVID-19 cross-border exposure events involving Germany which triggered a contact tracing-related activity of the RKI IC-team. Included were all activities with at least one confirmed case or contact person of a confirmed COVID-19 case and the start date of RKI event-specific activities between 3 February and 5 April 2020 (calendar weeks 6–14).

### Data analysis

The number of COVID-19 cross-border exposure events processed by the RKI during the study period was analysed to assess:

- number of German and international authorities in contact with the RKI and communication channels used;
- context and country of the exposure events as well as number of contacts followed up;
- time course according to country and context of exposure;
- time delay (time between last exposure and start of RKI activities) and time duration (time between start and end of RKI activities).

We used the following information for each event: date of the initial communication, communication channel, start and end date of the RKI activities, country which initiated the communication, number of national and international authorities the RKI was in contact with, country where the exposure occurred, date of the last possible exposure, context of the exposure, and number of contact persons.

The results were summarised graphically and descriptively using frequency counts (n), average (n), median (n), interquartile range (IQR; 25–75th percentile), range (min–max), and proportions (%). To determine differences between continuous variables, we used the Wilcoxon–Mann–Whitney test. The data analysis was conducted with Stata (software version 15, StataCorp).

Case studies of selected COVID-19 cross-border exposure events were used to highlight extent and complexity of cross-border contact tracing activities.

### Definitions

We made a distinction between countries where communication was carried out through EWRS in the EU/EEA and communication which occurred through the IHR NFP. Each authority with whom the RKI was in contact was counted once per event, the initially received information was not considered. The number of transport providers, travel agencies or other companies that provided data on contact persons and exposures, as well as communication with the Federal Ministry of Health (MoH), were not included in the analysis.

If no information on the country of exposure was available, the country which initiated the communication was used as the country of exposure. For events involving only contact persons in an aircraft or on a cruise ship, the country of exposure was indicated as ‘transnational’, since the exposure occurred during the journey. Possible means of transport included plane, ship, coach or taxi. For exposures not related to transport, a distinction was made between private overnight stays (e.g. hotel, guesthouse), professional events (e.g. congress, business meetings) and other social contexts (e.g. carnival, restaurant).

For larger groups, or if the last date of contact between the case and the contacts was unknown, the last date on which contact may have occurred (e.g. last day of a holiday, last day of a cruise) was used as the exposure date.

### Additional data source

Data available in Germany’s national reporting electronic database for communicable diseases (Surv*N*et@RKI) were also reviewed to supplement missing information on location and context of exposure for events notified within Germany [7].

### Ethical statement

The data were collected within the legal framework of the IfSG, the EU Decision 1082/2013 and the IHR. For the analysis, only aggregated data are presented. The information in the case studies was anonymised. Therefore, approval from an ethics committee was not sought.

## Results

Between 3 February and 5 April 2020 (calendar weeks 6–14), 467 cross-border contact tracing events with exposures in 30 countries were included in the analysis. The most frequent countries of exposure included Germany (n = 164; 35%), Austria (n = 101; 22%), Italy (n = 32; 7%) and Spain (n = 11; 2%). Outside the EU/EEA, Egypt, Israel and the United States were the most frequent countries (n = 5 each; 1%). The country of exposure was classified as transnational in 19% (n = 90). In 59% (n = 267) of all events, Germany initiated the communication and half of those events (n = 139) had also occurred there. In the case of 25 events, Germany was informed from abroad about an exposure in Germany.

The RKI IC-Team was in contact with different authorities 1,099 times. Of these, 55% (n = 600) were communications with German PHA, 31% (n = 345) were with EU/EEA countries via EWRS, 11% (n = 126) were with other countries via IHR NFPs and 3% (n = 28) were with the Federal Foreign Office or its embassies abroad. In 16 events, there was no contact to any authority because the contact person’s place of residence could not be determined. These persons were contacted directly by the RKI if an email address or a phone number was available. The median number of authorities the RKI was in contact with was 1 (IQR: 1–2) per activity (Table 1).

**Table 1:** Authorities the RKI was in contact with, contact persons and days of time delay, COVID-19 contact tracing, Germany, 3 February–5 April 2020 (n = 467)

	Number of events		Authorities the RKI was in contact with			Contact persons			Days of time delay ^a^	
	n	%	n	Median	IQR	n	Median	IQR	Median	IQR
**Total**	**467**	**100**	**1,099**	**1**	**1–2**	**5,099**	**2**	**1–6**	**8**	**5–11**
Country of initial communication ^b^										
Germany	276	59.1	643	1	1–2	3,135	2	1–5	8	5–12
Abroad	191	40.9	456	1	1–2	1,964	2	1–8	8	5–11
Country of exposure ^c^										
Germany	164	35.1	249	1	1–2	326	1	1–3	10	7–14
Abroad	213	45.6	452	1	1–2	1,894	2	1–7	7	4–10
Transnational	90	19.3	398	2	1–5	2,879	8	2–16	7	5–10

COVID-19: coronavirus disease; IQR: interquartile range; RKI: Robert Koch Institute.

^a^ Days of time delay were defined as time between last exposure of a contact person to a confirmed COVID-19 case and start of RKI activities.

^b^ Comparison of median number of authorities involved between communication initiated from Germany or abroad yielded a p value of 0.128. Comparison of median number of contact persons between communication initiated from Germany or abroad yielded a p value of 0.021. Comparison of median time delay between communication initiated from Germany or abroad yielded a p value of 0.528 (Wilcoxon–Mann–Whitney test).

^c^ Comparison of median number of authorities involved between exposure in Germany or abroad yielded a p-value of 0.439. Comparison of median number of authorities involved between exposure in Germany or transnational and abroad or transnational, respectively, yielded a p value of < 0.001. Comparison of median number of contact persons between exposure in Germany or abroad, Germany or transnational and abroad or transnational, respectively, yielded a p value of < 0.001. Comparison of median time delay between exposure in Germany or abroad and abroad or transnational, respectively, yielded a p-value < 0.001. Comparison of median time delay between exposure in Germany or transnational yielded a p value of 0.095 (Wilcoxon–Mann–Whitney test).

For 327 (70%) events, the exposure context was known. This included 93 (28%) events where the exposure occurred during transport (64 air travel, 24 cruise ships, five coaches/taxis/others). The remaining events included exposures in hotels or guesthouses (n = 83; 25%), at congresses, trade fairs and other business meetings (n = 64; 20%), or in other context such as private stays or gatherings (n = 63; 19%). In another 24 (5%) of the 327 events, several exposure contexts were mentioned. The median number of authorities contacted was highest for events with exposures related to cruises (Table 2).

**Table 2:** Cross-border contact tracing events for COVID-19, contact with health authorities and contact persons by exposure context, Germany, 3 February–5 April 2020 (n = 467)

Exposure context	Total number of cross-border contact tracing events		Number of authorities the RKI was in contact with															Number of contact persons ^b^		
			Total ^a^			German health authorities			EWRS			IHR NFPs			German Federal Foreign Office					
	n	%	n	Median	IQR	n	Median	IQR	n	Median	IQR	n	Median	IQR	n	Median	IQR	n	Median	IQR
**Total**	**467**	**100**	**1,099**	**1**	**1–2**	**600**	**1**	**0–1**	**345**	**1**	**0–0**	**126**	**0**	**0–0**	**28**	**0**	**0–0**	**5,099**	**2**	**1–6**
Aircraft	64	14	150	2	1–3	76	1	0–1.5	43	0	0–1	26	0	0–0.5	5	0	0–0	391	4	1–11
Cruise ship	24	5	247	10.5	2.5–16	190	9	2.5–12.5	29	0.5	0–1.5	14	0	0–1	14	0	0–1	2,467	60	9.5–269
Other means of transportation	5	1	9	2	1–2	5	1	1–1	4	1	0–1	0	0	0–0	0	0	0–0	2	1	1–1
Not transport-related event	210	45	398	1	1–2	220	0	0–1	136	1	0–1	38	0	0–0	4	0	0–0	1,510	2	1–6
Both transport- and not transport-related event	24	5	91	2	1–4	53	1	0.5–2.5	21	0	0–1	14	0	0–1	3	0	0–0	464	15	2–33
Unknown	140	30	204	1	1–2	56	0	0–1	112	1	0–1	34	0	0–0	2	0	0–0	265	1	1–2

COVID-19: coronavirus disease; EWRS: Early Warning and Response System; IHR NFP: International Health Regulations (2005) National Focal Point; IQR: interquartile range; RKI: Robert Koch Institute.

^a^ Comparison of median number of authorities involved between exposure on cruise ship or aircraft and cruise ship or not transport-related event, respectively, yielded a p value of < 0.001. Comparison of median number of authorities involved between exposure on cruise ship or other means of transportation yielded a p value of 0.019 (Wilcoxon–Mann–Whitney).

^b^ Comparison of median number of contact persons between exposure on cruise ship or aircraft and cruise ship or not transport-related event , respectively, yielded a p value of < 0.001. Comparison of median number of contact persons between exposure on cruise ship or other means of transportation yielded a p value of 0.245 (Wilcoxon–Mann–Whitney).

For 344 (74%) events, information on the number of contact persons was available. In total, data on 5,099 contacts was shared, with a median of two contacts (IQR: 1–6) per event. Fewer contact persons per event were involved when the communication was initiated in Germany or when the country of exposure was Germany rather than abroad or transnational (Table 1). Most contacts per event occurred on cruises (Table 2).

For 332 (71%) events, the date of exposure was known. The median time delay between date of exposure and receipt of the information by the RKI was 8 days (IQR: 5–11). The median time duration of RKI activities was 1 day (IQR: 1–3) and more among cruise-related exposures than for aircraft, other means of transport and not transport-related exposures (5 days (IQR: 3–9) vs 1 day (IQR: 1–4); p < 0.001, 3 days (IQR: 1–5); p = 0.255 and 1 day (IQR: 1–3); p < 0.001). For aircraft related exposures, the duration was longer for flights with arrival in Germany than for arrival abroad (median = 3 days (IQR: 1–6) vs 1 day (IQR: 1–3); p = 0.012).

**Figure 2:**
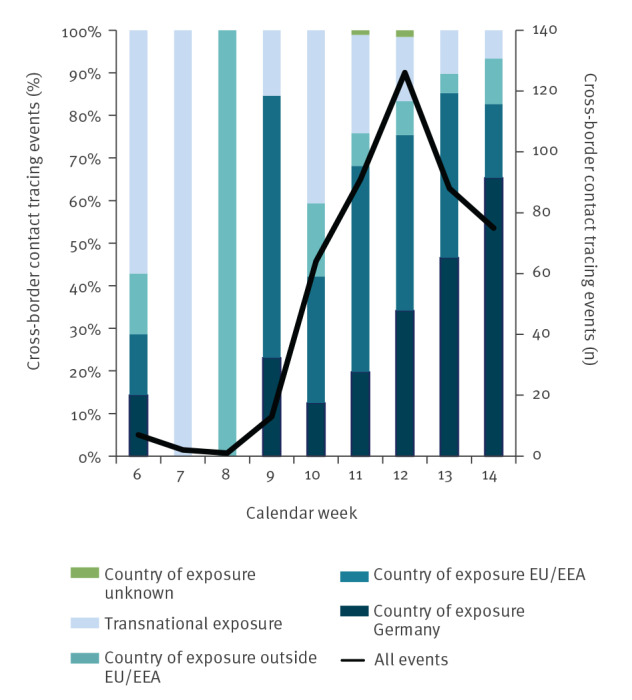
Number of cross-border COVID-19 contact tracing events and proportion of country of exposure, by week of activity start, Germany, 3 February–5 April 2020 (n = 467)

COVID-19: coronavirus disease; EEA: European Economic Area; EU European Union.

**Figure 3:**
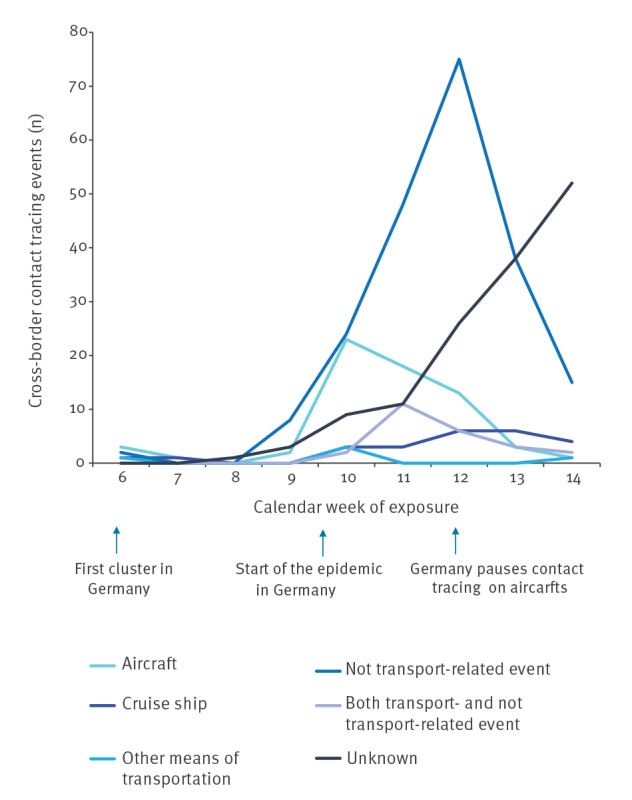
Number of cross-border COVID-19 contact tracing events, by exposure context and by week of activity start, Germany, 3 February–5 April 2020 (n = 467)

The total number of events increased starting in calendar week 9, peaked with 126 events in calendar week 12, and decreased to 75 events in calendar week 14. Detailed information regarding country and context of exposure is shown in Figures 2 and 3.

COVID-19: coronavirus disease.

The average number of people working per day on these activities increased steadily and showed a peak in calendar week 13 with seven people (range: 5–8). The increase in incoming information from February 2020 onwards required expansion of the team to accommodate the workload. Staff was recruited from all units of the Department for Infectious Disease Epidemiology at the RKI. To maintain high quality standards, training sessions as well as weekly team meetings and regular feedback groups were implemented.

### Case studies

The following selected case studies illustrate three of the major COVID-19 cross-border contact tracing events involving Germany.

In early March 2020, the RKI was informed by a German regional PHA of a person who 2 days prior had attended an international business meeting in Germany with 24 people. On the day after the meeting, the person was tested for SARS-CoV-2. When the positive test result arrived, the case had already travelled through Germany by train and taken a flight to Egypt. The respective IHR NFP was immediately informed of the case’s travel for contact tracing on the flight and known contacts in Egypt. Contact tracing for the train was not possible since the tickets for the neighbouring seats had been booked anonymously. The data on contact persons exposed during the business meeting were transmitted via EWRS to three EU/EEA countries. As the case also reported a holiday in South Tyrol, Italy in late February 2020 and onset of symptoms 4 days after the holiday 2020, information on fellow travellers was forwarded to the respective German federal states. Among them, three persons had become symptomatic in the meantime.

In early March 2020, the RKI was informed by a local PHA about two people who tested positive for SARS-CoV-2, one who had become symptomatic at the beginning of March 2020 and an asymptomatic fellow traveller. Both had taken part in a cruise with 45 guest cabins for 2 weeks in mid-February 2020 and spent a 1-week beach holiday afterwards. Subsequently, the IHR NFP Egypt was informed. The contact details of all persons participating in the cruise and residing at the hotel at the same time as the cases were collected and forwarded to the respective countries. Passengers seated in the same row as the confirmed cases or two rows in front and behind on the flight from Egypt to Germany were also investigated. Overall, data on 189 contacts residing in Germany were transmitted to 15 responsible health authorities. Moreover, five EU/EEA countries and one additional country outside the EU/EEA were informed of, respectively, 44 and nine contacts via EWRS and the IHR NFP.

The RKI was notified in late February 2020 by Italy about an Italian citizen who was tested SARS-CoV-2-positive and had attended a 3-day meeting in Munich with 13 close contact persons. The case became symptomatic 5 days before notification 2020. All participants had left Munich by the day after symptom onset of the case. Four contact persons had travelled by plane by the time of notification. One person took as many as seven flights. Data on contact persons was transmitted to Spain, France, the Netherlands, Denmark and Sweden via EWRS, as well as to the respective German local PHA. To our knowledge, eight of 13 contacts at the meeting were later tested SARS-CoV-2-positive.

## Discussion

This work describes cross-border contact tracing activities within Germany’s containment strategy of COVID-19 during the early stages of the pandemic [8], focusing on active case finding, early detection and isolation of cases and contacts in order to prevent and control the spread of infection. In addition, we presented case studies to exemplify the complexity of contact tracing owing to global mobility and multiple exposures during travel, business meetings or vacations.

The increase in cross-border contact tracing activities from calendar week 8 onwards reflects the increase of COVID-19 cases reported in countries outside of China [9]. The decrease after calendar week 12 can be attributed to international travel restrictions, the travel warnings issued by the German Federal Government and national travel-related quarantine measures. Worldwide increasing case numbers and limited personnel resources of local PHA as well as a pause of contact tracing on flights in Germany from 18 March 2020 may have also contributed to the decreasing number of incoming contact tracing activities after calendar week 12. Moreover, limited time for backtracking transmission routes may have led to the increasing number of events with an unknown exposure from calendar week 11 onwards. The proportion of events with reported exposure in Germany increased from calendar week 13 and reflects the increasing autochthonous case numbers in Germany.

The time delay from last possible exposure to start of RKI activities surpassed the median incubation period of COVID-19 [10,11]. This could be explained by the time it takes until a person seeks medical care, gets tested, is notified to the competent health authority and the initiation of investigations including forwarding of data to the RKI. Considering all these factors, such a time delay can be expected but still allows identifying potential secondary cases and interrupting transmission.

Travelling activities contributed to the geographical spread of SARS-CoV-2 infections [12-14] and therefore need to be considered as important events for contact tracing. While transport-related events did not represent the majority of the contact tracing events in our analysis, they were challenging and caused a long duration of RKI activities. Many international travellers in and from Germany generated numerous cross-border contacts in the transport sector. Moreover, the proximity between passengers due to limited space in public transport resulted in a large number of close contacts. Consequently, a large amount of personal data had to be distributed to different IHR NFPs, EU/EEA countries and/or local PHA in Germany. In this context, the communication with commercial transport companies and tour operators and timely receipt of complete personal data including contact details of passengers was one of the main challenges.

Air travel made up 69% of events in the category of transport-related exposures. In aircraft, passengers seated in the same row as a confirmed COVID-19 case and those seated two rows in front and behind were classified as close contacts. This cut-off was based on the assumed infection risk and has also been applied in the past for contact tracing in aircraft in the context of other infectious diseases transmitted by droplets [15-19]. Since passengers in aircraft usually remain seated, the number of contacts who need follow-up after air travel was smaller than for other means of transport. Still, the workload and consequently the duration of RKI activities was higher for flights with a destination in Germany as in this case, Germany was responsible for retrieving the passenger data from the airline.

After careful considerations, the RKI decided to pause contact tracing related to air-travel during the first peak of the COVID-19 pandemic in March 2020 because there was limited evidence of SARS-CoV-2 transmission on aircraft at that time and because human resources needed to be used efficiently. Until the end of the data collection period, SARS-CoV-2 transmission events on flights have been reported sporadically [20-23]. More conclusive studies on in-flight transmissions are needed to estimate the associated risk. In addition, currently implemented measures such as wearing of facemasks in aircraft need to be considered and evaluated. With the surge in travel activities after the relaxation of travel restrictions, Germany restarted contact tracing activities related to air travel exposure in calendar week 29 but adjusted its recommendation and defined as close contacts the persons seated directly next to a confirmed COVID-19 case; others seated in the two rows in front or behind were considered as contact persons with low-risk exposure.

That transmission among passengers and crew on cruise ships is particularly high has been reported in the literature [24-26]. In our analysis, the median number of contact persons related to ship travel was higher than that related to air travel. This can be explained by the long period of time passengers stay on the ship and movement and contacts associated with various activities such as restaurant visits, sports or entertainment. The large number of contact persons and the extensive communication, compared with other exposure contexts, led to a longer duration of RKI activities and a higher number of contacted stakeholders. The communication involved the German Federal Foreign Office and its embassies abroad and resulted in a long process of receiving and forwarding personal data of cases and contacts. Moreover, information on international travel routes of contact persons who had already disembarked had to be requested and followed up since the stay on a cruise ship was often only one part of a vacation route.

For many other means of transport including coaches and trains, conveyance operators do not systematically store passenger lists, seat reservations or the actual seat taken, which complicated the identification of close contacts. Because of the lack of standard procedures to handle passenger data, communication with conveyance and tour operators required a significant amount of time and human resources. Moreover, the completeness of the data varied widely across providers.

The effectiveness of communication with national and international PHA largely depended on the existing communication channels. The EWRS platform, which provides a single-window messaging system, facilitated direct communication with national PHA of EU/EEA countries and the United Kingdom as well as the safe transfer of personal data in compliance with the European Data Protection Regulation [6]. In contrast, communication with PHA in Germany and IHR NFP outside the EU/EEA required exchanging information by email and using an encrypted platform to share personal data. This was time-consuming and technically challenging since some recipients reported technical difficulties in retrieving data.

Owing to the federal structure of Germany, the legal responsibility of outbreak investigation lies with the municipal level. Within this task, RKI supported the PHA in international communication related to cross-border contract tracing during the COVID-19 pandemic. This led to a rapid increase of data exchange and adaption of the established communication routes with local PHA, which was a main challenge without a single-window messaging system. Moreover, missing information on exposure context, test date and symptoms of confirmed cases contributed to the duration of RKI activities. Still, the involvement of RKI as a national coordination point for international contact tracing activities was reported by local and regional PHA as a great support and an efficient way to pool and distribute relevant information on cross-border exposure events in time.

The data presented here were collected during the daily routine work of the RKI COVID-19 EOC and are thus subject to limitations. The number of contacts recorded did not reflect the total number of persons who had contact with a COVID-19 case in a cross-border context, since only information on contact persons related to Germany (e.g. residency or possible exposure in Germany) was processed by the RKI EOC. It was not possible to calculate accurate attack rates or distinguish between high- or low-risk contacts since data on the intensity of the contact, the number of contacts per COVID-19 case and the number of subsequently infected contacts was not available. Furthermore, the country or context of exposure was not always clear.

## Conclusion

Cross-border contact tracing in the current COVID-19 pandemic requires a lot of resources but is a critical component of the outbreak response in Germany and should be considered as one of the pillars of national preparedness and response strategies. A large pool of continuously trained staff and constant feedback can foster efficient workflows and helps spread the continuous high workload and knowledge to a sufficient number of qualified professionals to secure sustainability. Travel-related exposure events play an important role in cross-border contact tracing and associated challenges should be further addressed, including the need for efficient communication with all involved stakeholders. Consequently, national and international conveyance and tour operators should be legally obliged to store a minimal dataset of passenger data including name and contact details as well as seat or cabin information (as appropriate). Contact points for inquiries of competent PHA with provision of data within 24 h should be required. In addition, the implementation of a global platform for data exchange between the transport sector and PHA should be evaluated on an international level.

EWRS was perceived by the team as efficient for the rapid and secure international data exchange, especially in the face of the high data protection law requirements. A similar protected global platform should be established for IHR NFPs and within Germany to avoid unnecessarily time-consuming data exchanges. In addition, a standard protocol for minimal information requirements could streamline the national and international exchange of personal data.

For the future, an evaluation of the effectiveness of cross-border contact tracing, considering data and experiences from the perspective of an EU/EEA or WHO member state could help guide the most efficient strategies and lead to a harmonised international approach of cross-border contact tracing.
